# Fast Chemical Analysis of Droplets Unlocked by Ultra-Fast Ion Mobility Spectrometry

**DOI:** 10.1021/acs.analchem.5c05025

**Published:** 2025-10-07

**Authors:** Klaus Welters, Christian Thoben, Julius Schwieger, Alexander Nitschke, Tim Ostermeier, Stefan Zimmermann, Detlev Belder

**Affiliations:** a Institute of Analytical Chemistry, Leipzig University,Linnéstraße 3, Leipzig 04103, Germany; b Institute of Electrical Engineering and Measurement Technology, Leibniz University Hannover,Appelstr. 9a, Hannover 30167, Germany

## Abstract

This study presents the first coupling of high-throughput droplet microfluidics to an ultra-fast ion mobility spectrometer (IMS), providing a method for the label-free and comprehensive analysis of droplet contents at very high speeds. Building on the core strengths of IMS — compactness, simplicity, and cost-effectiveness — the added ability to capture full spectra at exceptionally short cycle times of less than 2 ms positions the technique as an exceptional platform for spectrometric analysis. The combination of a custom electrospray interface, a purpose-built monolithic fused-silica droplet generator chip, and an ultra-fast IMS enabled the chemical analysis of individual droplets in segmented flow at speeds of up to 120 Hz. The approach of droplet-based high-throughput ion mobility spectrometry (DHT-IMS) was also applied for reaction screening in nanoliter-sized droplets, exemplified by a hydrazone formation reaction between 5-methylisatin and phenylhydrazine.

## Graphical abstract

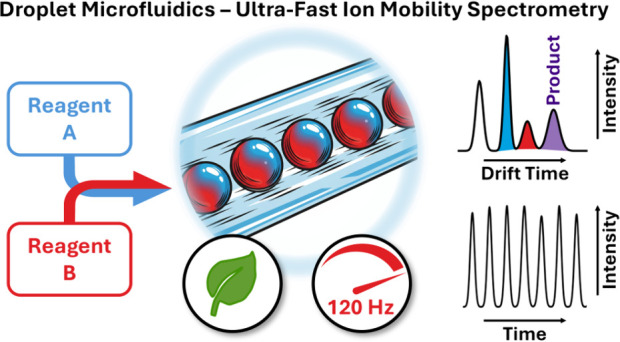

Microfluidic systems have established themselves as powerful platforms for miniaturized chemical processes, enabling a significant reduction in resource consumption while maintaining a high degree of automation and throughput.^1,2^ Among these, droplet microfluidics, which uses immiscible segmented flows to generate nanoliter- to picoliter-sized droplets, has proven particularly effective for compartmentalizing reactions and analyses.^3–5^ These droplets act as discrete microreactors that can be quickly manipulated, and thanks to their generation rates in the kilohertz range, they are particularly well-suited for high-throughput screening and high-content analysis.^6–8^

Despite this considerable potential, the analysis of chemically complex droplet contents at high frequencies remains a challenge. The standard technique for high-throughput droplet analysis is fluorescence detection,^9,10^ which offers exceptional sensitivity and detection rates. However, it suffers from limited chemical specificity and relies on the use of fluorophores, making it unsuitable for identifying unknown or unlabeled compounds. Alternative approaches - such as photothermal spectroscopy,^11^ Raman-based methods (including SERS)^12^ or UV-native fluorescence or absorbance^13,14^ - can somewhat expand the analysis range but still do not provide comprehensive chemical insights.

In this context, mass spectrometry (MS) has emerged as the most chemically informative detection technique compatible with droplet-based systems. MS enables detailed analysis of droplet contents, including unknown analytes and complex mixtures. However, its use in droplet microfluidics is limited by practical constraints: in particular, the limited detection rates (typically 50 Hz or less), the need for operation under high-vacuum conditions, and the related relatively high complexity and cost of the system severely limit its applicability, especially for high-frequency droplet streams. While recent instrumentational developments offer the potential to improve upon the throughput rates realized by MS,^15–17^ other limitations persist.

A promising and yet under-explored alternative is ion mobility spectrometry (IMS). IMS is an established, label-free gas phase separation technique that distinguishes ions based on their size, shape, mass and charge.^18,19^ Stand-alone IMS devices are particularly attractive for system integration due to their compact size, robustness, and high potential repetition rates, as they avoid the limitations of complex vacuum systems and the associated costs of MS. Although IMS is widely used in security, environmental and process monitoring,^20–24^ the potential of IMS for fast and chemically sensitive detection in microfluidic applications has only recently been explored.^25–28^

Building on recent advances in high-resolution IMS devices, including innovations in ion gating, signal processing and multiplexing,^29–35^ we have recently demonstrated for the first time the direct coupling of droplet microfluidics with a custom-built stand-alone IMS, thereby proving compatibility and analytical feasibility.^36^

With the recent development of an ultra-fast IMS system with repetition rates of up to 2000 Hz,^37^ it is now possible to match the speed of droplet formation in high-frequency microfluidics with chemically conclusive detection. This technological convergence opens a new avenue for real-time, label-free chemical analysis of fast-moving droplets without compromising throughput or information content.

In this study, we present the first integration of high-speed droplet microfluidics with ultra-fast IMS, demonstrating its application in droplet-based high-throughput (DHT) screening of chemical transformations. The work aims to evaluate both the capabilities and limitations of IMS as a detector for fast-moving droplet streams, thereby laying the foundation for future developments in real-time, marker-free analysis in microfluidic systems.

## Experimental Section

### Chemicals and Materials

Acetonitrile (ACN, ≥ 99,9%), methanol (MeOH, ≥ 99.9%) and 2-propanol (IPA, ≥ 99,8%) were purchased from VWR Int. GmbH (Darmstadt, Germany), while sodium acetate (NaAc, water-free) and tetrahexylammonium bromide (THA, 99%) were obtained from Merck KGaA (Darmstadt, Germany). 5-Methylisatin (>99.0%) and phenylhydrazine hydrochloride (>98.5%) were bought from TCI Europe N.V., (Zwijndrecht, Belgium). 3M Novec 7500 was sourced from IoLiTec-Ionic Liquids Technologies GmbH (Heilbronn, Germany), 008-FluoroSurfactant from RAN Biotechnologies, Inc. (Beverly, MA, USA). Water was purified using a TKA Smart2Pure purification system (<0.055 μS/cm, TKA GmbH, Niederelbert, Germany). Separate solutions of 5-MeI and PhH·HCl in MeOH, each at a concentration of 500 μM, were freshly prepared before measurements.

### Droplet Microfluidics

Discontinuous polar solvent-based droplets suspended in a continuous oil phase were produced using a monolithic fused silica (FS) droplet generator chip. The chip, shown in Figure 1A, was manufactured using selective laser-induced etching (SLE) (FEMTOprint aHead P2, FEMTOprint SA, Muzzano, Switzerland) from an FS wafer (Siegert Wafer GmbH, Aachen, Germany). All internal channels were designed with 100 μm internal diameters (IDs). Detailed information on the general workflows for fabricating chips using this method is provided elsewhere.^38^ The chip incorporated a Y-shaped connecting structure, allowing two streams to merge and form the discontinuous phase before droplet formation at a T-junction geometry. Both discontinuous and continuous phases were delivered to the droplet generator chip using fused silica capillaries (100 μm ID, 363 μm OD, Molex Polymicro TSP, Lisle, IL, USA). After their generation, droplets were transported from the chip via a transfer capillary made from perfluoroalkoxy alkane (PFA) with an approximate length of 30 cm (HPFA+ tubing, 100 μm ID, 360 μm OD, Postnova Analytics GmbH, Landsberg, Germany) to a custom-made nanoflow sheath liquid electrospray interface based on commercially available fittings and tubing (VICI AG, Schenkon, Switzerland). The central component of this sheath flow interface is a 380 μm ID, 1/16” OD stainless steel capillary, within which the PFA capillary was positioned concentrically. Both the stainless steel and the PFA capillary were tapered at the outlet ends by manual grinding to angles of approximately 10° and 20°, respectively, to form the emitter and facilitate spray formation. More detail and performance metrics of the interface are provided elsewhere.^39^ A commercial pressure-based flow controller system consisting of four individual pressure regulating units (Flow EZ 1000 mbar, Fluigent SAS, Le Kremlin-Bicêtre, France) and a corresponding control software (OxyGEN 2.3.2.0) was used to deliver both the discontinuous and the continuous phase as well as the sheath liquid for the droplet electrospray emitter. The flow of the continuous oil phase was regulated exclusively by pressure, while the flow rates of both discontinuous phase liquids and the sheath liquid were controlled using flow sensor units (Fluigent Flow Unit S) with custom calibration sets. Pressure and flow data were recorded for selected measurements using the software-integrated logging function.

**Figure 1:**
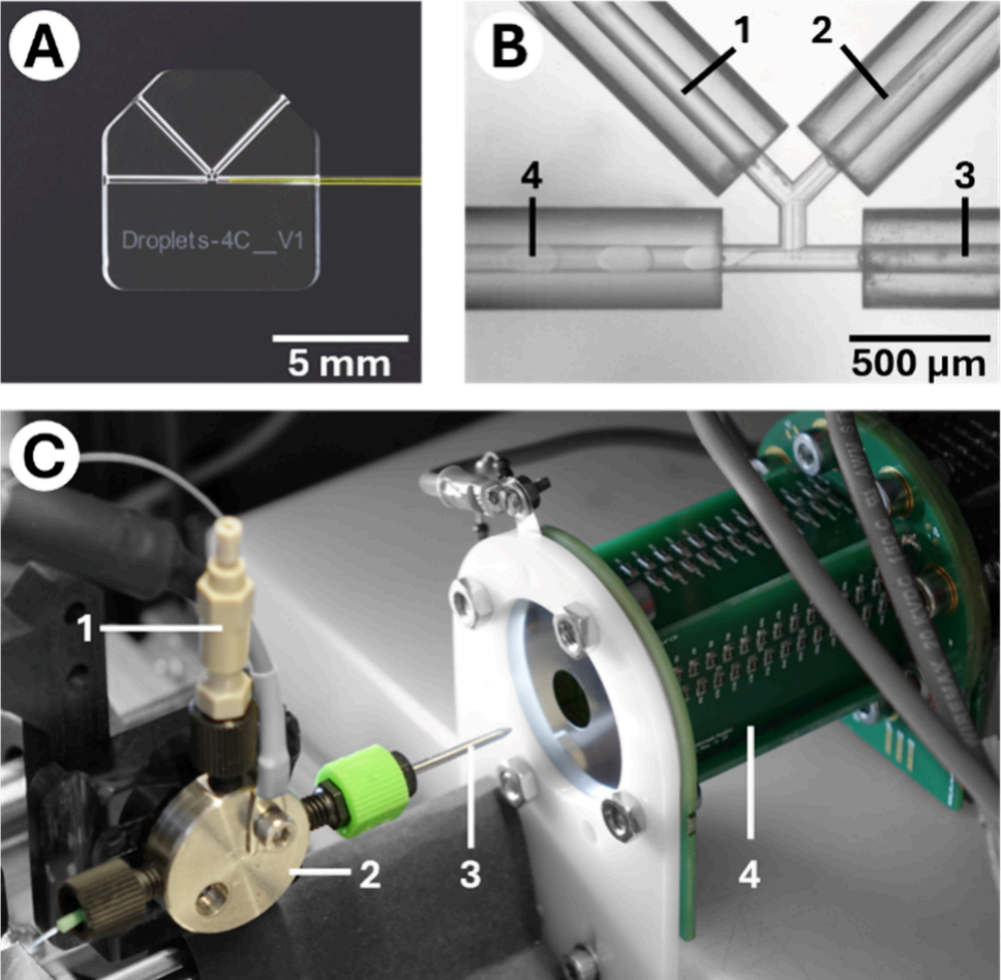
(A) Detailed view of the monolithic fused silica droplet generator chip. (B) Droplet generation process in the chip as captured by the high-speed camera. (1) Discontinuous flow channel 1. (2) Discontinuous flow channel 2. (3) Continuous flow. (4) Droplet transfer channel to IMS. (C) Droplet electrospray ionization interface coupled to the ultra-fast ion mobility spectrometer. (1) Sheath liquid supply line. (2) T-piece with high voltage connector. (3) Stainless steel emitter with enclosed droplet transfer capillary. (4) Printed circuit board-based ultra-fast IMS system with electrostatic inlet lens.

### Ion Mobility Spectrometry Instrumentation

The microfluidic droplet system was coupled to a compact ion mobility spectrometer. High voltage for the electrospray was supplied by an external power supply (HCL 35–6500, FuG Elektronik GmbH, Schechen, Germany) and was applied between the emitter and the grounded inlet of the desolvation region. Therefore, the detector and the amplifier as well as the ADC were at a high potential of up to 12.5 kV. A detailed description of the isolated data acquisition is given elsewere.^33^ The IMS is a printed circuit board (PCB)-based drift tube instrument capable of very fast repetition rates, up to 2 kHz, when utilizing helium as the drift gas.^37^ A self-designed and self-constructed isolated DC power supply with 50 kV isolation and a high overall efficiency of 82.5% at 55 W is used to supply the electronics.^40^ An overview of the instrument and operating parameters is given in Table 1 and a photo of the setup is shown in Figure 1C. A more extensive description of the instrument, including an optional setup with commercially available components for providing the voltage sequence of the center grid of the tristate ion shutter, is given elsewhere.^37^ The IMS was controlled via a LabVIEW application (Version 2018, National Instruments, Austin, TX, USA). IMS raw data was filtered using a 15 kHz low-pass filter and subsequently processed using MATLAB (version R2022a, Mathworks, Natick, MA, USA) and/or OriginPro 2019 (Origin Lab Corporation, Northampton, MA, USA). A detailed list of the filtering applied to data sets is provided in Table S1. In this work, all measurements were carried out in positive ion mode.

**Table 1:** IMS Instrument Operating Parameters

desolvation voltage	–6.0 kV	drift gas flow rate	240 mL/min
drift voltage	–6.5 kV	drift gas	N_2_
desolvation length	50 mm	pressure	1001–1004 hPa
drift length	34 mm	temperature	297–304 K
injection time	5–40 μs		

### High-Speed Videography

To assess the frequency, size, and spacing of the generated droplets, high-speed recordings of the droplets were taken at the beginning of the transfer capillary, immediately after generation on the fused silica droplet generator chip, shown in Figure 1B. A Phantom VEO 410L camera (Vision Research Inc., Wayne, NJ, USA), capturing 10,000 frames per second at a resolution of 832 × 600 pixels, was used. Employing an NDPL-1­(2x) ocular adapter, the camera was mounted on a microscope equipped with a 4x objective (Bresser GmbH, Rhede, Germany) that was modified by the addition of a custom-made actively cooled 3300 lm COB LED light source (JU1215-KM309, Everlight Electronics Co. Ltd., Taipei, ROC).

## Results and Discussion

We demonstrated the successful development of an analytical platform for droplet monitoring using ultra-fast IMS, capable of full-spectrum, continuous droplet characterization at rates up to 120 Hz. This platform builds upon previous work of hyphenating droplet microfluidics to IMS and incorporates recent advancements in IMS instrumentation. The results presented here highlight the feasibility and advantages of applying this approach to ultra-high throughput screening, with a projected capacity exceeding 10^7^ droplets per day.

### Initial Evaluation of Droplet-Based High Throughput Analysis via Ion Mobility Spectrometry

To demonstrate a proof of principle, droplets containing 50 μM of tetrahexylammonium (THA) in a 1/1 (v/v) mixture of ACN/H_2_O with added 1 mM NaAc were analyzed using a matching 8/2 (v/v) ACN/H_2_O sheath liquid at droplet rates of up to 31 Hz (Figure 2). These initial experiments were successful, revealing a periodically fluctuating analyte signal, with intense peaks each corresponding to the infusion of individual droplets. This assignment was further supported by high-speed videography, which showed a high level of agreement between the generated droplets and the corresponding measured IMS data. The sheath flow rate was optimized based on the spray cone shape, ESI quality and IMS signal stability, with the exact conditions shown in Table S2. The combination of a monolithic fused silica droplet generator chip, the custom-made ESI interface, and the ultra-fast IMS allowed for a high quality of both droplet generation and measured signal, yielding residual standard deviations (RSDs) of 5.2% and 4.9% for (droplet) peak area and height in the THA measurements (Figure 2) at 31 Hz. For these experiments injection times of 5 μs were used, which yielded spectra with a resolving power (fwhm) of *R*_p_ = 80 for the THA peak. The system demonstrated a high degree of repeatability over multiple days, with the *K*_0_ value measured for THA (0.888 cm^2^/V·s) exhibiting an RSD of only 0.73%.

**Figure 2:**
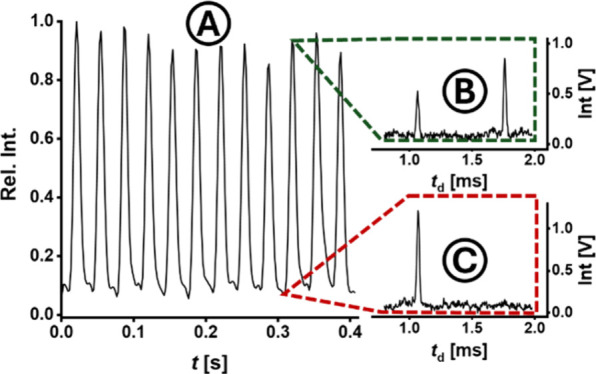
Proof of principle measurement of droplet-based high-throughput analysis for droplets at a generation rate of 31 Hz. Droplet composition: Discontinuous phase: 50 μM tetrahexylammonium bromide (THA) in 1/1 (v/v) ACN/H_2_O with added 1 mM NaAc. Continuous phase: 0.5% (w/v) 008-Fluorosurfactant in Novec 7500. Sheath liquid: 2.0 μL/min 8/2 (v/v) ACN/H_2_O. (A) Signal amplitude of the drift time of 1.77 ± 0.06 ms, specific for the analyte THA. Individual droplets can be identified as the analyte is only contained in the droplets. Full ion mobility spectra recorded for every data point in the chromatogram show signals for either both solvent and analyte (B) at drift times of 1.07 and 1.77 ms for droplets or only signal for solvent (C) for the continuous phase, with the solvent signal stemming primarily from the sheath liquid.

### High-Speed Droplet Detection: Achievements and Considerations

To further exploit the potential of droplet microfluidics, subsequent experiments were conducted using an in-droplet reaction. A condensation reaction between 5-methylisatin and phenylhydrazine, yielding a hydrazone product (Figure 4A) served as the model system. Similar reactions have been previously investigated for reaction optimization in charged microdroplets, though typically at higher concentrations and delivered in a continuous sample stream.^41,42^ Here, the chemistry served primarily to assess the droplet system’s detection capabilities rather than for reaction optimization. Each reagent, dissolved in pure MeOH, was introduced separately through the channels 1 and 2 of the Y-shaped merging structure on the chip (Figure 2B). This ensured that the reagent streams combined only immediately before droplet formation. To promote efficient ionization, the sheath liquid was adjusted to an 8/2 (v/v) MeOH/H_2_O mixture. Furthermore, comparison experiments using direct infusion ESI of a premixed reagent mixture were performed. With both sample composition and device operating parameters adjusted to accommodate for the higher amount of sample supplied by direct infusion compared to droplets, the results showed strong agreement between the spectral information obtained from direct infusion (Figure S1) and droplet experiments (Figure S2). This confirmed the compatibility of droplet microfluidics with the established analytical process. Based on this confirmation, the system was tested with progressively higher droplet speeds (21 Hz shown in Figure 3A), equaling those demonstrated in the previous experiment. Exemplary micrographs of droplets, their size and spacing at different generation speeds are provided in Figure S4 and Table S3. The injection time was increased to 40 μs for the ensuing droplet experiments. Consistent with literature,^37^ this afforded a distinct increase of signal amplitude and a slight decrease of resolving power of *R*_p_ = 60 for the product peak. Focused on exploring the system’s limitations and leveraging the scalability of a droplet-based system, the droplet generation rate was drastically increased.

**Figure 3:**
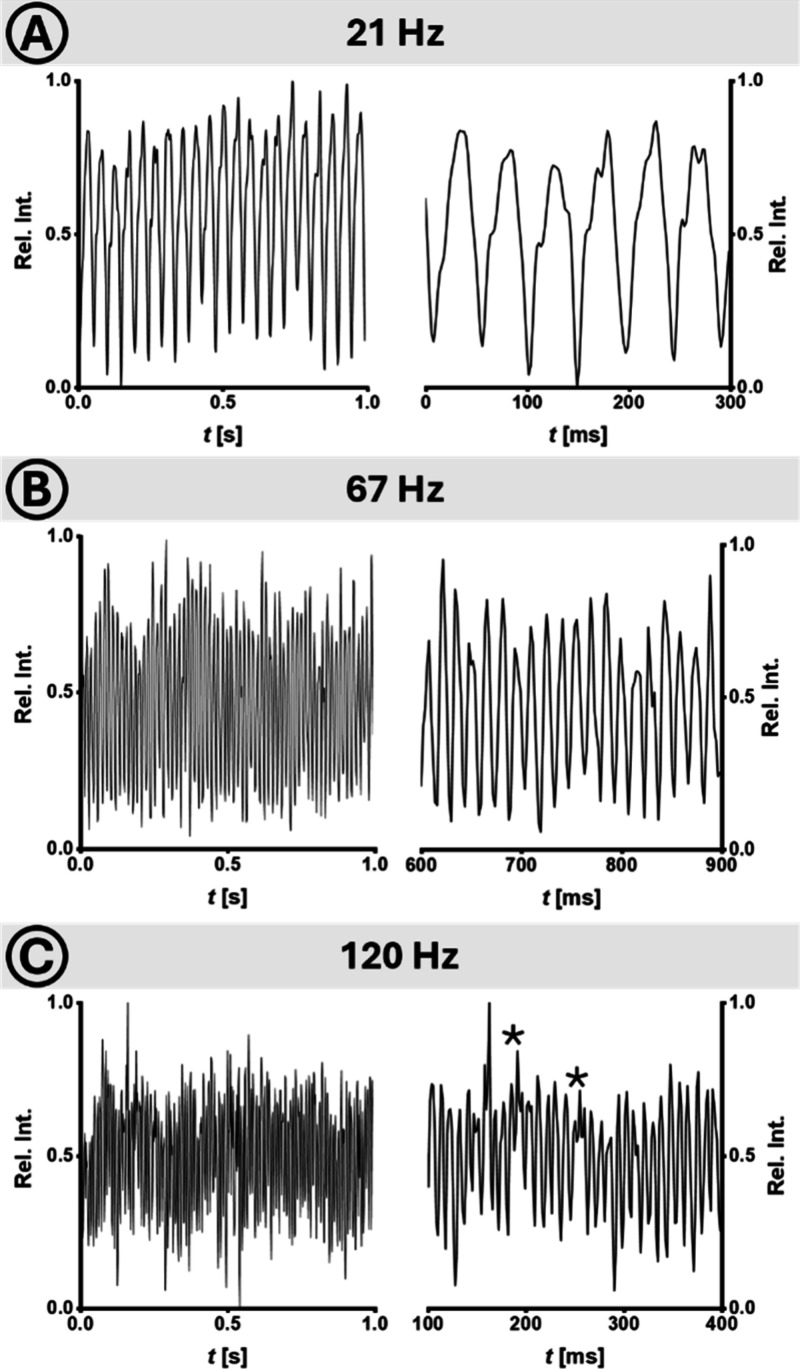
Ultra-high throughput droplet measurements. Droplet composition: Discontinuous phase: 250 μM each phenylhydrazine and 5-methylisatin in MeOH. Continuous phase: 0.5% (w/v) 008-Fluorosurfactant in Novec 7500. Sheath liquid: 0.3–1.0 μL/min 8/2 (v/v) MeOH/H_2_O. Signal amplitudes of the base peak, each shown at a time scale of 1.0 s and a detail at 300 ms, for droplet generation at approximately (A) 21 Hz, (B) 67 Hz and (C) 120 Hz. The peaks marked with asterisks (C) have approximately double the width and area of the neighboring peaks, presumably indicating droplet coalescence.

Eventually, droplet rates unprecedented by both IMS and MS were reached, with successful detection demonstrated for 67 Hz (Figure 3B), other intermediate rates, but most notably for a rate of 120 Hz (Figure 3C). A corresponding high-speed video is available in the Supporting Information. The ability to operate at such high rates provides significant advantages, including finer temporal resolution of dynamic processes and greater efficiency in screening and reaction monitoring. While substantial increases in droplet rates generally led to a decline in signal quality (Table S1), individual droplets could still be identified by characteristic peak shape patterns present in all tested speeds. At droplet rates greater than 67 Hz signals presumably caused by droplet coalescence could be observed. The decline in signal quality at higher droplet rates is consistent with literature^17^ and is likely linked to limitations of the ionization process at the ESI interface emitter tip. Higher droplet rates correlated with increased flow, exceeding 5 μL/min, which typically necessitates the use of a supporting nebulizer gas. However, the use of a sheath or nebulizer gas would interfere with the drift tube IMS’s outflowing constant gas stream. As such, the system relied on an increased ESI voltage to achieve effective nebulization. This approach, while effective, can lead not only to corona discharges but also to distortions and irregular droplet behavior,^43^ even before reaching the emitter tip. Overcoming these constraints - and potentially enabling even higher droplet rates - will require interface designs capable of handling a wide range of flow rates while maintaining stable, reproducible electrospray for each droplet. Although adjusting the spray voltage and emitter position can accommodate a surprisingly broad flow range, further reductions in the internal diameters of capillaries and chips offer a promising route. While this approach involves challenges - such as maintaining robustness and securing suitable capillaries and pumping systems - it also has the potential to significantly reduce sample consumption. Conversely, the detection system must be capable of acquiring a sufficient number of data points across each analyte peak. Given the similarity between the droplet trace pattern and a chromatogram, it is reasonable to take guidance from criteria applied in chromatography. Recent publications in the field suggest that only six data points per peak may be sufficient even for quantitative analysis.^44^ This, in turn, has direct effects on the applicable acquisition settings. For most experiments a maximum drift time of two milliseconds was monitored. This resulted in a repetition rate of 500 Hz, theoretically sufficient for droplet rates up to 83 Hz. To afford measurements at a droplet rate of 120 Hz (Figure 3C), the maximum monitored drift time was reduced to 1.6 ms, increasing the effective repetition rate to 625 Hz. While this does not inherently reduce sensitivity, significantly larger ions with higher drift times may be excluded. The repetition rate could be further increased by raising the drift potential, while using helium as the drift gas could allow rates up to 2000 Hz for certain species, potentially tripling the droplet speeds demonstrated in this work.

### Real-Time Reaction Monitoring and Stoichiometric Control in High-Throughput Experiments

Building on the results of the high-speed droplet experiment, the system was employed for high-throughput screenings for droplets with varying compositions of the reaction shown in Figure 4A: Since the reagent mixing occurred immediately prior to droplet formation, precise control over the composition of individual droplets was achieved by adjusting the relative flow rates of the reagent streams. As a result, variations in reagent concentrations could impact both product yield and ionization efficiency. Analyzing the signals of both educts and product via IMS enabled real-time and continuous monitoring, facilitating the potentialy seamless optimization of stoichiometric ratios. To demonstrate this, the initial educt ratio of 4:1 - delivered by the discontinuous flow channels 1 and 2 - was inverted for 10 s and then returned to its original state, while maintaining a constant total flow across both channels. (Figure 4B, flow values shown in Figure S3). The corresponding IMS data (Figure 4C) enabled the precise determination of both the residence time and the droplet composition. After a residence time of 29.3 s, the effect of inverting the educt ratio became apparent after a transient time of only 0.35 s, closely aligning with pressure readings recorded by the pumping system, indicating a near-instantaneous response of the droplet generation system. The reaction-in-droplet experiment with variable reagent ratios was conducted at a rate of 23 Hz, as this allowed for an overall good signal quality while retaining a very high rate of potential sample throughput per day. For instance, in the interval of 35.0 to 36.2 s shown in Figure 4, RSDs of approximately 6% were obtained for both droplet peak area and height. At this droplet rate fewer than 10 droplets with vague composition are generated, markedly reducing the quantities of reagents and solvents consumed during these brief equilibration transients. This efficiency underscores the suitability of DHT-IMS to satisfy the demands put forward by green chemistry approaches, further supported by a calculated greenness score of 0.86 (Figure 5), based on the AGREE metric system.^45^ Based on these results, DHT-IMS appears as a highly promising candidate for fully automated reaction screening approaches, offering precise control over droplet composition and the capability to generate large numbers of droplets for each composition, while maintaining excellent resource and energy efficiency.

**Figure 4:**
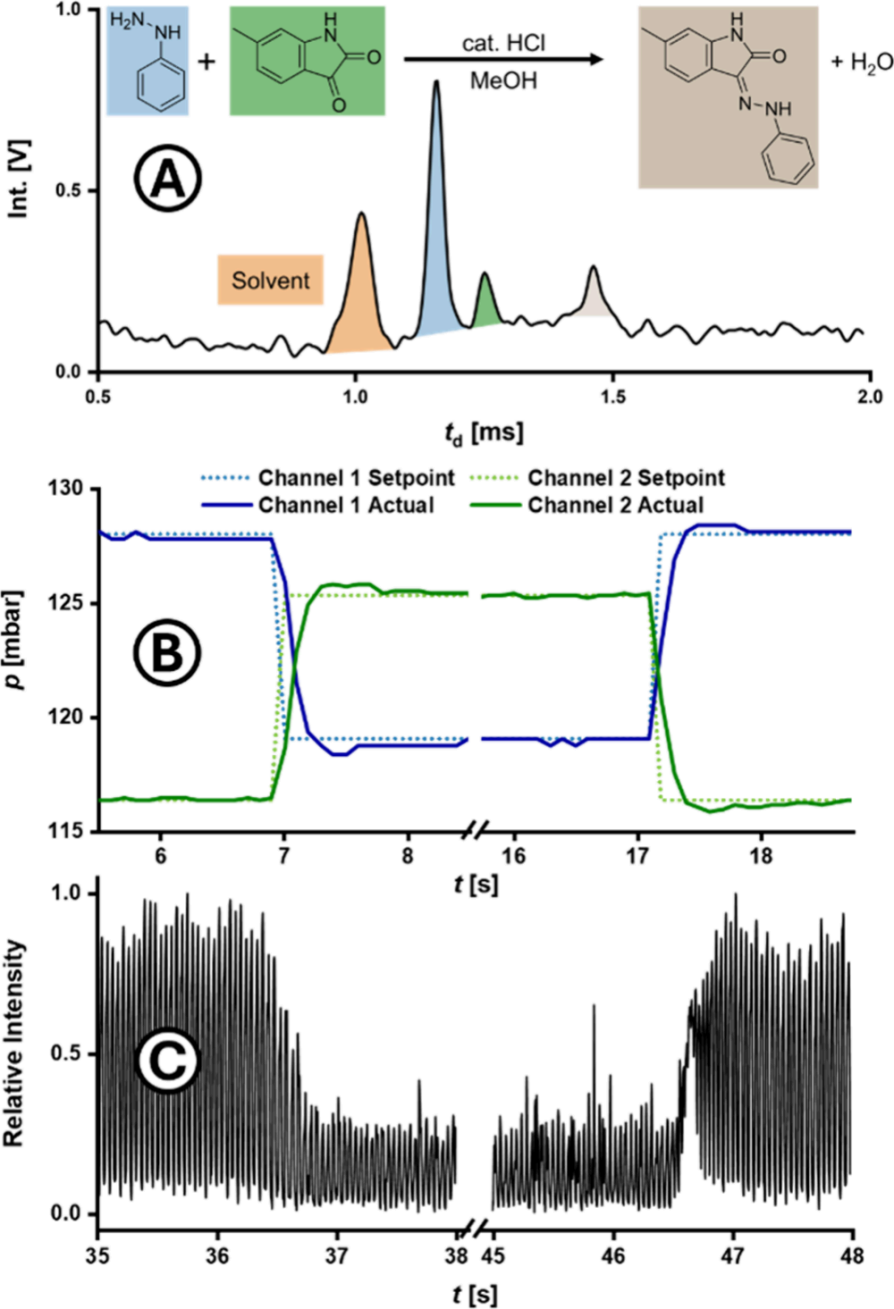
Low latency dosing adjustment for variation of droplet composition demonstrated by a reaction-in-droplet experiment at 23 Hz. Discontinuous phase: Varying concentrations of phenylhydrazine and 5-methylisatin in MeOH. Continuous phase: 0.5% (w/v) 008-Fluorosurfactant in Novec 7500. Sheath liquid: 1.0 μL/min 8/2 (v/v) MeOH/H_2_O. (A) Reaction scheme and ion mobility spectrum in-droplet (peak at 36.162 s in (C)). Assigned peaks for solvent (1.01 ms, orange), phenylhydrazine (1.15 ms, blue), 5-methylisatin (1.25 ms, green) and the reaction product (1.47 ms, taupe). (B) Pressure set points and actual readout values of the pump channels supplying the discontinuous phases. Channel 1 supplied phenylhydrazine, channel 2 5-methylisatin. (C) Signal amplitude of the drift time of 1.15 ± 0.06 ms, specific for phenylhydrazine, indicating strong agreement of droplet composition as analyzed per IMS with that predicted from flow-controlled pump rates shown in (B).

**Figure 5:**
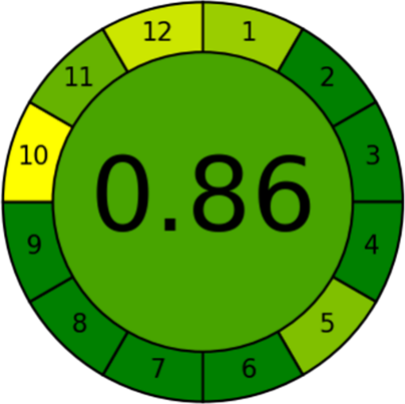
Greenness assessment diagram for DHT-IMS generated using the AGREE metric system and corresponding software.

## Conclusions

This work presents, to the best of our knowledge, the fastest spectrometric analysis method applied to droplet microfluidics to date, employing ion mobility spectrometry to achieve continuous, full-spectrum analysis of droplets at high speed, reaching droplet rates of up to 120 Hz. The presented results demonstrate the high potential and broad possibilities of IMS as a robust and cost-effective detector for droplet microfluidics, enabling high-throughput analysis at a miniaturized scale. The combination of those techniques can deliver comprehensive chemical information for the content of every droplet at speeds previously unattainable by either mass- or ion mobility spectrometry.

Building on the past achievements of combining droplet microfluidics with IMS, as well as the recent introduction of a novel superfast IMS, this work demonstrates the successful application of this hyphenation for ultra-high-throughput methodologies. Satisfying demands of green chemistry approaches such as a greatly reduced consumption of solvents and high energy efficiency, a theoretical sample throughput of more than 10^7^ droplets per day, all equaling potential individual samples per day, is achievable.

## Supplementary Material





## Notes

Additional ion mobility spectra for comparative direct infusion ESI-IMS measurement and droplet measurements, flow and pressure rate recordings, list of applied data filtering, ionization conditions and flow rates for each experiment shown, micrographs of droplet generation, estimated droplet spacing and sizing (PDF)
High-speed video of the droplet generation at 120 Hz (MP4)

## Notes

Klaus Welters – Institute of Analytical Chemistry, Leipzig University, Linnéstraße 3, 04103 Leipzig, Germany

## Notes

Christian Thoben – Leibniz University Hannover, Appelstr. 9a, 30167 Hannover, Germany

## Notes

Julius Schwieger – Institute of Analytical Chemistry, Leipzig University, Linnéstraße 3, 04103 Leipzig, Germany

## Notes

Alexander Nitschke – Leibniz University Hannover, Appelstr. 9a, 30167 Hannover, Germany

## Notes

Tim Ostermeier – Leibniz University Hannover, Appelstr. 9a, 30167 Hannover, Germany

## Notes

Stefan Zimmermann – Leibniz University Hannover, Appelstr. 9a, 30167 Hannover, Germany
