# Supplementary material for: Fast Chemical Analysis of Droplets Unlocked by Ultra-Fast Ion Mobility Spectrometry
Source: Anal Chem. 2025 Oct 7;97(41):22932–8. doi: 10.1021/acs.analchem.5c05025 (PMC12547850; doi:10.1021/acs.analchem.5c05025)
Supplement: Supplementary file 1 [file ac5c05025_si_001.pdf]

SUPPORTING INFORMATION

Fast Chemical Analysis of Droplets Unlocked by Ultra-Fast Ion Mobility Spectrometry

Klaus Welters [a], Christian Thoben [b], Julius Schwieger [a], Alexander Nitschke [b], Tim Ostermeier [b], Stefan Zimmermann [b], Detlev Belder [a]*

[a] *Institute of Analytical Chemistry, Leipzig University, Linnéstraße 3, 04103 Leipzig, Germany*

[b] *Institute of Electrical Engineering and Measurement Technology, Leibniz University Hannover, Appelstr. 9a, 30167 Hannover, Germany*

Corresponding author:

*E-mail Detlev Belder: belder@uni-leipzig.de

Table of contents

Figure S1	–	Direct infusion ESI-IMS measurement of the model reaction	page – S2
Figure S2	–	Ion mobility spectra of droplet and non-droplet	page – S2
Figure S3	–	Pressure and corresponding flow rates	page – S3
Figure S4	–	Micrographs of droplet generation	page – S4
Table S1	–	List of filters applied for data processing	page – S3
Table S2	–	List of spray voltages, discontinuous and sheath flow rates	page – S4
Table S3	–	List of estimated droplet volume and spacing.	page – S4
Video S1	–	High-speed video of droplet generation at 120 Hz	

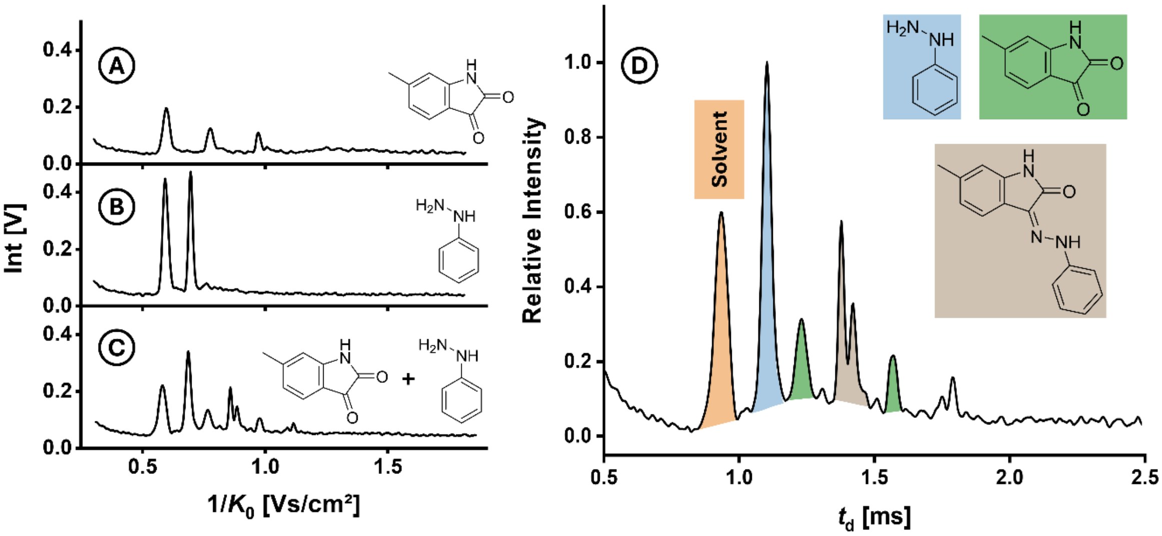

Figure S2. Direct infusion (non-droplet) ESI-IMS experiment of the hydrazone formation reaction. (A) 100 µM 5-methylisatin, (B) 100 µM phenylhydrazine hydrochloride and (C) a mixture of 100 µM each in 100% MeOH. The peaks are assigned in (D). The peak at ca. 1.6 ms is presumably a dimer of 5-methylisatin, as is already observable for the pure compound in (A). ESI conditions: 40/363 µm ID/OD tapered fused silica emitter, 1 µL/min, +3.0 kV spray voltage. IMS settings: 5 µs injection time, 20 kHz low pass filter, 20 spectra averaged, positive ion mode. $1/K_0$ values were calculated according to the following formula using the drift time (t_d), the drift region length (L) potential (ΔU) as well as temperature (T) and pressure (p) that were measured by device-integrated sensors: $1/K_0 = ((L^2 \cdot p \cdot T)/(\Delta U \cdot t_d \cdot p_0 \cdot T))^{-1}$.

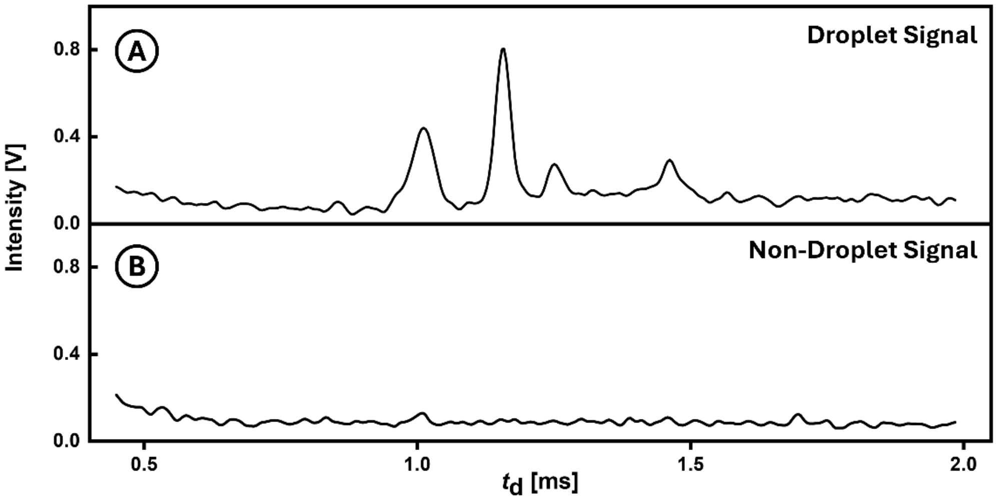

Figure S1. Ion mobility spectra of (A) high intensity (discontinuous phase / droplet signal, 36.162 s) and (B) low intensity (continuous phase / non-droplet signal, 36.184 s) regions of the chromatogram shown in Fig. 3C for in-droplet reaction at 23 Hz. Discontinuous phase: Varying concentrations of PhH·HCl and 5-MeI in MeOH. Continuous phase: 0.5% (w/v) 008-Fluorosurfactant in Novec 7500.

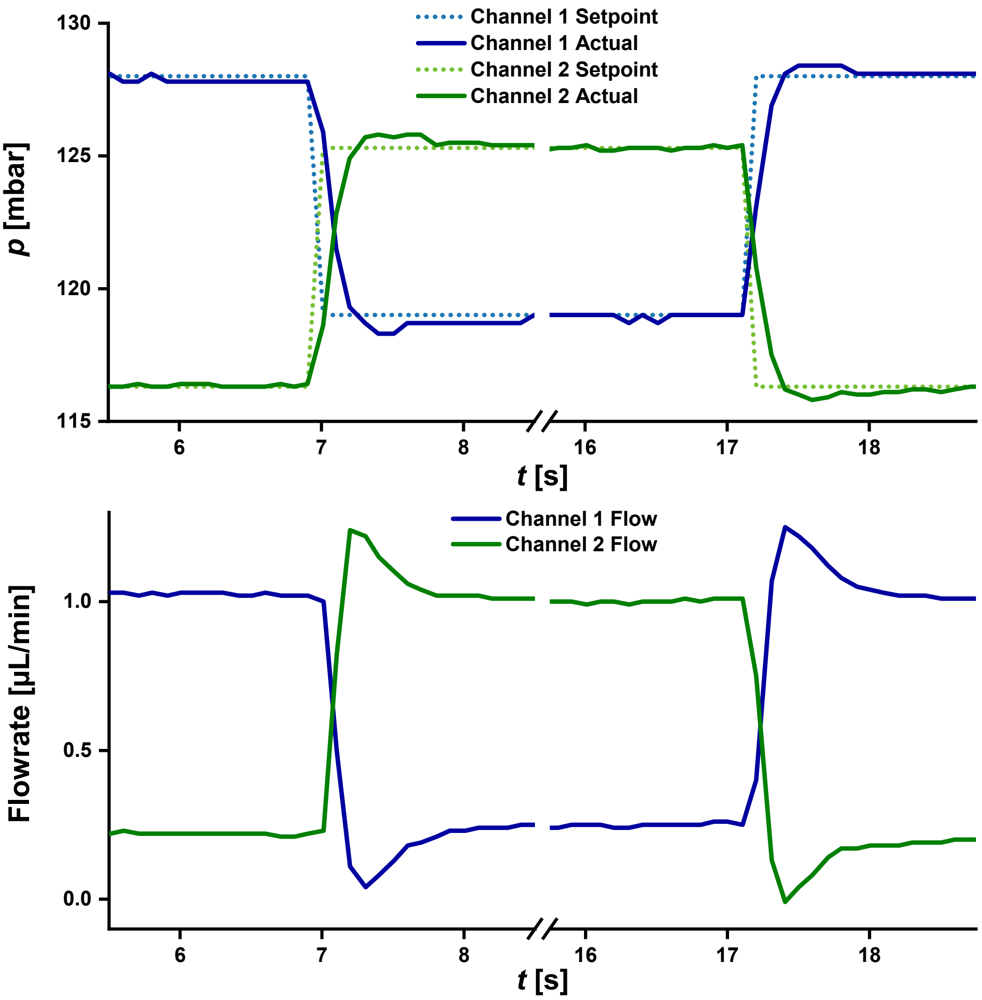

Figure S3. Pressure and flow rates as recorded by the commercial pressure-based pumping system control software for the experiment with variable reagent concentrations. An initial overshoot in flow rate, typical of transient behavior in pressure-based systems, is visible. Employed in all experiments were Flow EZ 1000 mbar controllers, Flow Unit S sensors and OxyGEN 2.3.2.0 control software (all from Fluigent).

Table S1. List of applied filtering and number of droplet peaks used for calculation of RSDs of peak height and areas.

Experiment / Droplet Rate	FFT Filter f_{cutoff} / Hz	n_{RSD}	RSD_{Height} / %	RSD_{Area} / %
THA 31 Hz	125	24	4.86	5.19
Rct 21 Hz	125	31	8.01	8.97
Rct 67 Hz	250	33	15.5	13.0
Rct 120 Hz	312.5	27	22.3	25.0
VarRct 23 Hz	125	25	6.53	6.36

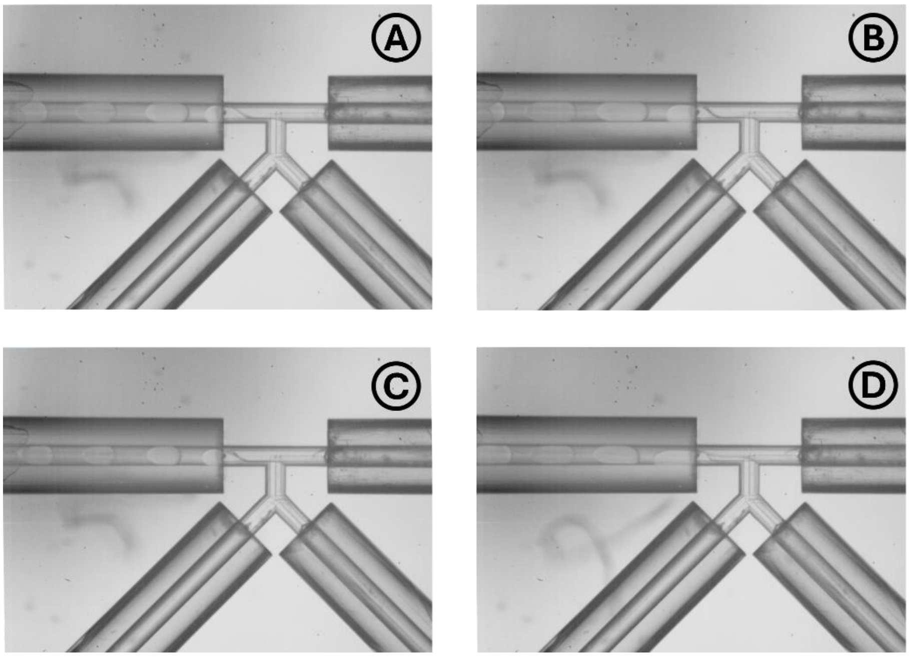

Figure S4. Screenshots of video capture of droplet generation at different speeds. An estimation of droplet spacing is provided below. (A) 15 Hz (B) 37 Hz (C) 60 Hz (D) 120 Hz.

Table S2. List of experiments with corresponding conditions of spray voltage, and flows of sheath liquid and discontinuous phases.

Experiment / Droplet Rate	Voltage ESI [kV]	Sheath Flow [nL/min]	Channel 1 Flow [nL/min]	Channel 2 Flow [nL/min]
THA 31 Hz	2.5	2000	/	/
Rct 21 Hz	3.0	1000	340	330
Rct 67 Hz	4.5	1000	1070	1130
Rct 120 Hz	5.5	300	3000	3700
VarRct 23 Hz	4.5	1000	200 – 1000	200 – 1000

Table S3. Estimated droplet spacing.

Rate	$V_{Droplet}$ / nL	$V_{Droplet}$ RSD / %	Ratio l_{disc}/l_{cont}
15 Hz	1.24	6.4	1.37
37 Hz	1.64	3.4	2.45
60 Hz	1.17	2.2	1.01
120 Hz	1.30	3.9	1.97

Video S1.

Droplet generation at 120 Hz – provided on the publisher's website.
